# Cardioprotective function of sclerostin by reducing calcium deposition, proliferation, and apoptosis in human vascular smooth muscle cells

**DOI:** 10.1186/s12933-023-02043-8

**Published:** 2023-11-02

**Authors:** Sheila González-Salvatierra, Cristina García-Fontana, Jesus Lacal, Francisco Andújar-Vera, Luis Martínez-Heredia, Raquel Sanabria-de la Torre, María Ferrer-Millán, Enrique Moratalla-Aranda, Manuel Muñoz-Torres, Beatriz García-Fontana

**Affiliations:** 1 Biosanitary Research Institute of Granada (ibs.GRANADA), Granada, 18012 Spain; 2 Endocrinology and Nutrition Unit, University Hospital Clínico San Cecilio, Granada, 18016 Spain; 3 Department of Biochemistry and Molecular Biology II, University of Granada, Granada, 18071 Spain; 4 Institute of Health Carlos III, CIBER of Frailty and Healthy Aging (CIBERFES), Madrid, 28029 Spain; 5 Laboratory of Functional Genetics of Rare Diseases, Department of Microbiology and Genetics, University of Salamanca (USAL), 37007 Salamanca, Spain; 6 Institute of Biomedical Research of Salamanca (IBSAL), 37007 Salamanca, Spain; 7 Bioinformatic Research Service, Biosanitary Research Institute of Granada (ibs.GRANADA), Granada, 18012 Spain; 8 Department of Computer Science and Artificial Intelligence, University of Granada, Granada, 18071 Spain; 9 Andalusian Research Institute in Data Science and Computational Intelligence (DaSCI Institute), Granada, 18014 Spain; 10 Department of Biochemistry, Molecular Biology III and Immunology, University of Granada, Granada, 18071 Spain; 11 Nuclear Medicine Unit, University Hospital Clínico San Cecilio, Granada, 18016 Spain; 12 Department of Medicine, University of Granada, Granada, 18016 Spain; 13 Department of Cell Biology, University of Granada, Granada, 18016 Spain

**Keywords:** Type 2 Diabetes, Cardiovascular Diseases, Atherosclerosis, Sclerostin, Vascular smooth muscle cells, Protective role

## Abstract

**Background:**

Sclerostin is an inhibitor of the Wnt/b-catenin pathway, which regulates bone formation, and can be expressed in vascular smooth muscle cells (VSMCs). Type 2 diabetes (T2D) is associated with an increased risk of cardiovascular disease (CVD) and increased serum and tissue expression of sclerostin. However, whether the role of sclerostin is detrimental or protective in the development of CVD is unknown. Therefore, our aims are to determine the level of sclerostin in T2D patients with/without CVD and in controls, both at serum and vascular tissue, and to analyze the role of sclerostin in VSMCs under calcified environments.

**Methods:**

Cross-sectional study including 121 controls and 139 T2D patients with/without CVD (48/91). Sclerostin levels in serum were determined by ELISA, and sclerostin expression was analyzed by RT-qPCR and immunohistochemistry in calcified and non-calcified artery of lower limb from T2D patients (n = 7) and controls (n = 3). *In vitro* experiments were performed in VSMCs (mock and sclerostin overexpression) under calcifying conditions analyzing the sclerostin function by determination of calcium and phosphate concentrations, and quantification of calcium deposits by Alizarin Red. Proliferation and apoptosis were analyzed by MTT assay and flow cytometry, respectively. The regulation of the expression of genes involved in bone metabolism was determined by RT-qPCR.

**Results:**

A significant increase in serum sclerostin levels in T2D patients with CVD compared to T2D patients without CVD and controls (*p* < 0.001) was observed. Moreover, higher circulating sclerostin levels were independently associated with CVD in T2D patients. Increased sclerostin expression was observed in calcified arteries of T2D patients compared to non-calcified arteries of controls (*p* = 0.003). *In vitro *experiments using VSMCs under calcified conditions, revealed that sclerostin overexpression reduced intracellular calcium (*p* = 0.001), calcium deposits (*p* < 0.001), cell proliferation (*p* < 0.001) and promoted cell survival (*p* = 0.015). Furthermore, sclerostin overexpression exhibited up-regulation of *ALPL* (*p* = 0.009), *RUNX2* (*p* = 0.001) and *COX2* (*p* = 0.003) and down-regulation of inflammatory genes, such as, *IL1β* (*p* = 0.005), *IL6* (*p* = 0.001) and *IL8* (*p* = 0.003).

**Conclusions:**

Sclerostin could play a protective role in the development of atherosclerosis in T2D patients by reducing calcium deposits, decreasing proliferation and inflammation, and promoting cell survival in VSMCs under calcifying conditions. Therefore, considering the bone-vascular axis, treatment with anti-sclerostin for bone disease should be used with caution.

**Graphical Abstract:**

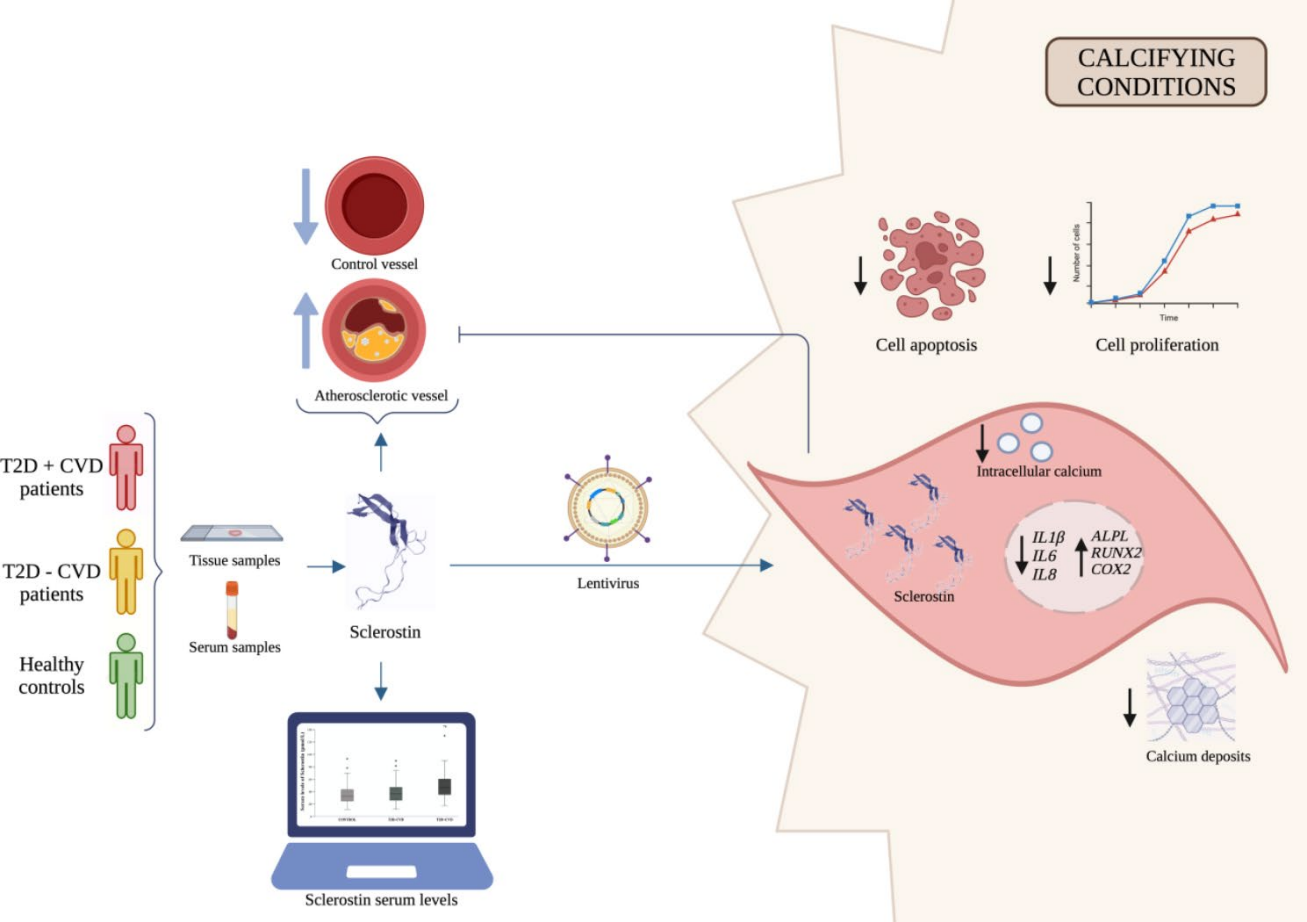

## Background

Type 2 diabetes (T2D) is associated with an increased risk of cardiovascular disease (CVD) [1] affecting approximately 35% of T2D patients [2]. The major CVDs associated with T2D include ischemic heart disease, coronary artery disease, and peripheral artery disease [2]. Atherosclerosis is the main pathological mechanism underlying CVD in T2D, due to hypertension, hyperglycemia and dyslipidemia [3]. The development of atherosclerosis is strongly associated with the proliferation and migration of vascular smooth muscle cells (VSMCs) and endothelial activation [4]. To repair vascular damage in atherosclerosis, VSMCs switch from the contractile phenotype (characterized by high levels of contractile protein production) to synthetic phenotype (characterized by an increase in the production of cytokines and extracellular matrix). This facilitates proliferation and mobility as a physiological response induced by proinflammatory stimuli and oxidative stress [5, 6]. Moreover, VSMCs are capable of undergoing a phenotypic transition to osteocyte-like cells in a calcifying microenvironment capable of expressing typical osteocyte markers [7], as occurs in patients with T2D [8]. At this point, the Wnt/b-catenin pathway plays a crucial role in regulating VSMCs proliferation, migration and survival via modulation of the expression of matrix proteins [5, 9]. Proteins involved in the Wnt/b-catenin pathway, such as WNT4 and dickkopf-related protein, have been shown to stimulate the proliferation of VSMCs causing intimal thickening in atherosclerosis [10, 11]. Therefore, inhibitors of the Wnt/b-catenin pathway may facilitate the development of therapeutic targets for the treatment of CVD.

Sclerostin, a protein synthesized by the *SOST* gene, is an inhibitor of the Wnt/b-catenin pathway that regulates bone formation [12]. Under physiological condition, sclerostin is mainly expressed by osteocytes [13], although it is also expressed by VSMCs in calcifying environment [7]. In the serum level, sclerostin had been found highly expressed in T2D patients with atherosclerotic lesions [3, 14, 15]. Also, several studies have shown an association between sclerostin levels in serum and the occurrence of CVD and cardiovascular mortality [12, 16–18]. These findings strongly support that sclerostin, in addition to regulating bone metabolism, is involved in vascular homeostasis, constituting an important modulator of the Wnt/b-catenin pathway in CVD. Sclerostin has also been found highly expressed in calcified aortic tissue derived from human aortic samples from patients with atherosclerosis [19]. Sclerostin expression in the tunica media of VSMCs in plaques from patients undergoing carotid endarterectomy, suggests a role in the development of atherosclerosis [20]. A recent study in mice showed that calcifications in the aortic medial layer and renal vessels were significantly more pronounced when warfarin treatment was combined with anti-sclerostin antibody treatment, suggesting a protective effect of sclerostin during vascular calcification [21]. Despite these important advances, no human studies have demonstrated whether sclerostin plays a detrimental or protective role in atherosclerotic disease. Currently, a humanized monoclonal anti-sclerostin antibody, is being used in the clinical practice for the treatment of osteoporosis and high fracture risk [22]. Likewise a recent evaluation of this treatment has shown benefits on bone but an potential increased rate of adverse cardiovascular events [23–25]. These findings suggest that sclerostin inhibition could be associated with cardiovascular risk, although the role of this protein in CVD has not yet been clarified in humans. It is therefore crucial to clarify the role of sclerostin in pathophysiological vascular mechanisms in order to prevent and reduce the high morbidity and mortality rate associated with CVD.

This study aims to investigate the potential protective role of sclerostin in the development of atherosclerosis in humans, which has not been previously explored. To accomplish this, we pursued two primary objectives. Firstly, we quantified sclerostin levels in serum and vascular tissue samples obtained from individuals with T2D, both with and without CVD, as well as from control subjects. This analysis allowed us to assess the association between sclerostin and atherosclerosis in a clinical context. Secondly, we conducted experiments using sclerostin in VSMCs exposed to calcified environments in vitro. This approach enabled us to examine the direct impact of sclerostin on VSMCs under conditions relevant to atherosclerosis development. By combining clinical and experimental investigations, our study aims to provide comprehensive insights into the potential protective role of sclerostin in atherosclerotic processes, thereby contributing to a deeper understanding of the pathophysiology of this condition in humans.

## Methods

### Study population

This cross-sectional study included 260 participants, 121 healthy controls (65 ± 9 years, 56.2% males) and 139 T2D patients (65 ± 8 years, 56.1% males). T2D was diagnosed according to American Diabetes association criteria [26]. Healthy controls were recruited from Nuclear Medicine Unit at the University Hospital Clínico San Cecilio of Granada (Spain) between 2020 and 2022. T2D patients, on the other hand, were recruited from the Endocrinology and Nutrition Unit of the same hospital between 2017 and 2018. In both groups, recruitment was based on specific criteria, including Caucasian ethnicity and normal values for blood count, and hepatic function. These rigorous criteria ensured the selection of suitable individuals for the study, minimizing potential confounding factors and enhancing the reliability of the research findings. T2D group was classified in two subgroups according to the presence of CVD: CVD group (n = 48) and non-CVD group (n = 91). Inclusion criteria for CVD were cerebrovascular disease (transient ischemic attack or ischemic stroke), coronary heart disease (previous myocardial infarction, angina diagnosed with stable or unstable coronary artery bypass graft surgery), or ischemic peripheral arterial disease. Patients with hepatic, gastrointestinal, thyroid or bone diseases and those with an estimated glomerular filtration rate (eGFR) below 45 mL/min/1.73m^2^ or treated with thiazolidinediones, warfarin or other drugs that affect to bone metabolism were excluded.

Vascular tissue samples were obtained from artery of lower limbs at the Angiology and Vascular Surgery Unit of the University Hospital Clínico San Cecilio of Granada. Calcified vessels were obtained from T2D patients with ischemic diabetic foot with criteria of critical ischemia, in whom major lower limb amputation was indicated because they were not candidates for revascularization (primary amputation) or because it had failed (secondary amputation) (n = 7). The sample obtained was a fragment (10 mm) of the distal third of the superficial femoral artery. The stiffer or even stenotic or occluded arterial segment with visible atherosclerotic plaque was the one extracted for the research study. Samples of non-calcified vessels without any visible atherosclerotic lesions proceed from healthy subjects with no history of vascular risk factors or any ischemic event at any level with informed consent at the University Hospital Clínico San Cecilio of Granada (n = 3).

The Biobank of the Andalusian Public Health System at the University Hospital Clínico San Cecilio of Granada was responsible for the management of all samples used in this study. Prior to participation, informed consent was obtained from each subject, ensuring their voluntary involvement. The study was conducted in accordance with the guidelines and regulations set forth by the Ethics Committee of the University Hospital Clínico San Cecilio of Granada, and it adhered to the principles outlined in the World Medical Association Declaration of Helsinki (Project ID:0858-N-17. Research Ethics Committee of Granada Center at 26th April 2017).

### Clinical evaluation of study population

The height, weight, and waist circumference were measured at baseline according to standard procedures. The body mass index (BMI) was calculated by the Queletet formula, weight (kg)/stature (m^2^). The systolic and diastolic blood pressures were measured using a standard electronic sphygmomanometer. Hypertension was defined as values 140/90 mmHg and/or antihypertensive treatment. Dyslipidemia was characterized by serum levels of low-density lipoprotein cholesterol (LDL-c) > 100 mg/dL, high-density lipoprotein cholesterol (HDL-c) < 50 mg/dL, triglycerides (TG) > 150 mg/dL, and/or current treatment with lipid-lowering drugs. Patients reported their alcohol use, smoking status, and level of physical activity was recorded using the Spanish version of the questionnaire for Rapid Assessment of Physical Activity [27].

### Biochemical measurements of study population

Samples of venous blood were taken in the morning after fasting overnight. Serum samples were stored at -80ºC until analysis at the Clinical Analysis Unit of the University Hospital Clínico San Cecilio of Granada. The parameters as fasting plasma glucose (FPG), glycated haemoglobin (HbA1c), TG, HDL-c, LDL-c, phosphorus, and calcium were measured using standard automated laboratory techniques. eGFR was calculated using the Chronic Kidney Disease Epidemiology Collaboration equation (CKD-EPI) [28].

The calciotropic hormone profile included serum intact parathormone (iPTH) was determined by the two-site immunoassay for iPTH (Roche Diagnostics) and 25-hydroxyvitamin D (25(OH)D) was determined using a chemiluminescence immune assay (CLIA) (Beckman Coulter UniCel DxI 800). Total osteocalcin (OC) was determined by CLIA (N-Mid Osteocalcin; Immunodiagnostic Systems iSYS automated analyzer). The procollagen type 1 N-terminal propeptide (P1NP) and serum carboxy-terminal crosslinked telopeptide of type I collagen (CTX) were determined by electrochemiluminescence immune assay (ECLIA) (Roche Diagnostics). The alkaline phosphatase (ALP) levels were measured by a colorimetric method in an AU5800 analyzer (Beckman Coulter). Sclerostin and periostin levels were determined by the enzyme-linked immunosorbent assay (ELISA) method, following the manufacturer’s protocols (Biomedica). Precision testing was performed by the determination of intra-assay and inter-assay variations for each ELISA assay (5% and 1% for sclerostin; 6% and 3% for periostin).

### RNA isolation and RT-qPCR

RNA isolation and RT-qPCR were performed to quantify the expression of sclerostin in vascular tissue, to check the efficiency of transduction in primary human aortic smooth muscle cells (HAoSMCs) with lentiviral particles, and to quantify the expression of different genes under different conditions. For vascular tissue, total RNA was obtained by isolation of 23 transversal sections (3 μm) of calcified lower limb artery of T2D patients with peripheral artery disease and of non-calcified lower limb artery from healthy donors. RNA extractions were carried out with Trizol Reagent (ThermoFisher Scientific) by a manual homogenizer. For cells, RNA was isolated using a RNeasy Mini Kit (QIAGEN) according to the manufacturer’s instructions. In both cases, RNA was treated with Turbo DNase (Ambion), and the RNA concentration and quality were assessed using the Qubit Flex Fluorometer (ThermoFisher Scientific). Only RNA samples with a A260/280 ratio between 1.8 and 2.0 were used for cDNA synthesis.

The RNA was reverse-transcribed to synthesize cDNA using the iScript cDNA synthesis kit (BioRad), following the manufacturer’s protocol.

qPCR was carried out using PowerUp SYBR Green Master Mix (ThermoFisher Scientific) in a QuantStudio™ 7 Flex Real-Time PCR System (ThermoFisher Scientific) as follows: 95 °C for 2 min, 40 cycles of 95 °C for 20 s and 65 °C for 20 s. The analysis of the melting curve was performed from 65 to 95^o^C with increment of 0.5^o^C/4 sec. Primers were designed using the Primer Blast software (NCBI) and Clone Manager Suite program (Table 1). The expression of a constitutive gene was used to normalize the mRNA data. All real-time PCR reactions for each sample were performed in triplicate. Relative expression of each gene of interest was assessed using the 2^−ΔΔCt^ method [29].

**Table 1:** Primers used in the different experiments of this study

Gene	Sequence (5´-3´)		Amplicon (pb)	Application
*SOST*	Forward	ATGCCACGGAAATCATCCCC	185	Expression in vascular tissue. Check the efficiency of cells transduction. Quantify the expression under different conditions in VSMCs in vitro.
	Reverse	GTCACGTAGCGGGTGAAGTG		
*RPL13*	Forward	CGTAAGATCCGCAGACGTAAGGC	228	Constitutive gene
	Reverse	GGACTTGTTCCGCCTCCTCGGAT		
*ALPL*	Forward	GGCTGGAGATGGACAAGTTC	152	Quantify the expression under different conditions in VSMCs in vitro.
	Reverse	ACGCTCAGTGGCTGCGCTTA		
*RUNX2*	Forward	CGCCGTGGTCCTATGACCAGTCTTA	169	Quantify the expression under different conditions in VSMCs in vitro.
	Reverse	AGGCAGAAGTCAGAGGTGGCAGTGT		
*IL1β*	Forward	CTTCAGGCAGGCCGCGTCAGTTGTT	202	Quantify the expression under different conditions in VSMCs in vitro.
	Reverse	CCGGAGCGTGCAGTTCAGTGATCGT		
*IL6*	Forward	AGACAGCCACTCACCTCTTCAGAAC	208	Quantify the expression under different conditions in VSMCs in vitro.
	Reverse	CCAGGCAAGTCTCCTCATTGAATCC		
*IL8*	Forward	GAGAGTGATTGAGAGTGGACCAC	112	Quantify the expression under different conditions in VSMCs in vitro.
	Reverse	CACAACCCTCTGCACCCAGTTT		
*COX2*	Forward	CCGCCATCATCCTAGTCCTCATCGC	143	Quantify the expression under different conditions in VSMCs in vitro.
	Reverse	TAGTCCGCCGTAGTCGGTGTACTCG		
*ACTA2*	Forward	CATCGTGCTGGACTCTGGAGATGGT	151	Quantify the expression under different conditions in VSMCs in vitro.
	Reverse	GAAGGAATAGCCACGCTCAGTCAGG		

*SOST*, sclerostin; *RPL13*, ribosomal protein L13; *ALPL*, alkaline phosphatase, biomineralization associated; *RUNX2*, runt-related transcription factor 2; *IL1β*, interleukin 1 beta; *IL6*, interleukin 6; *IL8*, interleukin 8; *COX2*, cyclooxygenase 2; *ACTA2*, actin aortic smooth muscle; VSMCs, vascular smooth muscle cells.

### Immunohistochemistry and immunofluorescence of vascular tissue

Formalin-fixed paraffin-embedded biopsy tissues from calcified lower limb artery of T2D patients and non-calcified of healthy controls were obtained from archival paraffined in slides. The samples were deparaffined using a standard protocol by a combination of xylenes and ethanol. In order to perform the antigen retrieval, the tissue sections were incubated in 1X citrate buffer inside a steamer machine at high temperature (200 °C) for 20 min.

For immunohistochemistry, tissue sections were then rinsed in phosphate-buffered saline (PBS) (0.01 M, pH 7.4), incubated for 15 min with 3% hydrogen peroxide, rinsed again, and incubated in a solution of 3% normal goat serum and 0.1% PBS-tween20 for 60 min. Slices were transferred to an anti-sclerostin primary antibody (1:500, #ab85799, Abcam) solution overnight at 4ºC. After being rinsed with PBS, the sections were incubated with a secondary antibody (1:5000 Goat Anti-Rabbit IgG (H&L) HRP, #ab205718, Abcam) solution for 120 min at room temperature. Primary and secondary antibody solutions were mixed in a solution of 3% normal goat serum and 0.1% PBS-tween20. The sections were rinsed for further processing using the ABC-kit (Vector Laboratories). The reaction was visualized using the peroxidase substrate kit DAB (Vector Laboratories). Finally, the sections were rinsed, rehydrated with ethanol and xylenes and cover slipped. Images of lower limb artery of T2D patients and controls were captured using a light microscope (Olympus BX41). Slices containing the regions of interest were identified using the *Stereo Investigator* Software (mbf Bioscience) from coronal sections of the samples (Fig. 4A). In each sample, 4 microphotographs were captured at 20X magnification (Fig. 4B). The quantification of sclerostin was obtained using the *Image J* Software. For each microphotograph threshold objects (black circular dots over the white background) having specific size (35–150 µm^2^) and circularity (0.35-1.00) values matching those positive nuclei were automatically identified by the software as proteins. In order to equalize all the microphotographs and to cancel out possible background noise, they were previously converted into 8-bit type image and the background was lightened (150.0 pixels). The threshold was set up to 0-150 for all images.

For immunofluorescence, tissue sections were then rinsed in PBS (0.01 M, pH 7.4) and incubated in a solution of 3% normal goat serum and 0.1% PBS-tween20 for 60 min. Slices were transferred to an anti-sclerostin primary antibody (1:200; #ab85799, Abcam) solution overnight at 4ºC. After being rinsed with PBS, they were incubated in a secondary antibody (1:500, Goat Anti-Rabbit IgG (H + L) cross-adsorbed Secondary Antibody, Alexa fluor 488, #A-11008, Thermo Fisher) solution for 120 min at room temperature. Primary and secondary antibody solutions were mixed in a solution of 3% normal goat serum and 0.1% PBS-tween20. The sections were washed four times for 10 min in PBS and were mounted on glass slides with Fluoroshield Mounting Medium with 4’,6-diamidino-2-306 phenylindole (DAPI) (#ab104139, Abcam) and cover-slipped. The slices were then kept in the dark at 4°C and later observed using an upright Olympus BX41 confocal microscope. In each sample microphotographs at 20X magnification were captured using the blue (DAPI) and green (sclerostin) filters. The quantification of sclerostin (green circular dots) and cell nuclei (blue circular dots) were performed using the *Image J* Software. Representative microphotographs of the different groups are shown in Fig. 4D.

### Cell culture and reagents

HAoSMCs (ATCC) were grown using Vascular Cell Basal Medium (ATCC) supplemented with VSMC Growth Kit (ATCC) that contains the following components: recombinant human (rh) fibroblast growth factor (FGF-b), rh insulin, ascorbic acid, L-glutamine, rh epidermal growth factor (EGF), and fetal bovine serum (FBS). Cells were grown to confluence and used from passages 4 to 5.

Human embryonic kidney 293T cells (HEK293T) (ATCC) were cultured in DMEM/F-12 GlutaMAX (Gibco) supplemented with 10% FBS (NeoBiotech RNase A (Powder)).

Both cell cultures were grown under standard conditions (37 °C and 5% CO_2_ in a humid atmosphere).

### Second-generation lentiviruses and transduction for generation of stable lines of HAoSMCs

Overexpression of sclerostin was carried out by producing a second-generation lentiviral packaging system protocol using the vectors pVSV-G (Addgene), that expresses the envelope gene of the VSV-G virus, psPAX2 (Addgene) that expresses the reverse transcriptase gene, the protease gene and the gene for assembly of the HIV-1 virus and, the pLVX:*SOST* construct or empty pLVX (Addgene). HEK293T cells were transfected with the mix of the above plasmids using polyethylenimine (Quimigen) and were cultured in DMEM/F-12 GlutaMAX (Gibco) supplemented with 10% FBS and grown under standard conditions for 24 h. Lentivirus particles were harvested, filtered, ultracentrifuged, resuspended in PBS and stored at -80ºC.

HAoSMCs were transduced using lentivirus particles with polybrene infection reagent 8 mg/mL (Merck) and selected with Hygromycin B 50 mg/mL (ThermoFisher Scientific). Control cells (mock) were transduced with lentiviruses generated from the empty pLVX vector. Transductions were performed in triplicate and cells were cultured under standard conditions using Vascular Cell Basal Medium supplemented with VSMC Growth Kit. Finally, sclerostin overexpression in this stable cell line was tested by RT-qPCR.

### Induction of calcification

Transduced HAoSMCs with sclerostin overexpression and mock were seeded on 6-well plates at a confluency of 1000 cells/well. The cells were maintained in growth medium supplemented with 1.5 mM CaCl_2_ and 10 mM β-glycerophosphate for up to 20 days to induce matrix calcification. Incubation cells were grown under standard conditions and the medium was changed every second/third day.

### Calcium measurement

Extra- and intracellular calcium concentration was determined by the Calcium Colorimetric Assay Kit (BioVision). Extracellular calcium was measured from the culture medium. Cells were treated with 0.1 M NaOH and 0.1% Sodium Dodecyl Sulfate (SDS) to measure intracellular calcium. Quantification of calcium was measured with an optical density (OD) at 570 nm by spectrophotometry. The results were normalized to the total protein concentration, which was measured in the cultures using a protein assay reagent (Bio-Rad), based on the Bradford dye binding procedure, and albumin was used as a standard. Each condition was performed in triplicate and expressed as µg calcium/µg protein.

Additionally, calcium mineral deposition was assessed by Alizarin Red S staining. Cells were washed twice with PBS, fixed in 4% paraformaldehyde for 30 min at room temperature, rinsed with distilled water 3 times, stained with 2% alizarin red (pH 4.2) for 30 min with gentle shaking at room temperature and the dye was removed and cells were washed 5 times with distilled water. Alizarin red stained cultures were extracted with 10% acetic acid and incubated at room temperature for 30 min with shaking. Cells were collected in a microcentrifuge tube and vortexed. The tubes were heated at 85°C for 10 min, incubated on ice for 5 min and centrifuged. 10% ammonium hydroxide was added, then the OD of the dissolved dye was measured at 405 nm spectrophotometrically. The results were normalized to the total protein concentration, which was measured in the cultures using a protein assay reagent (Bio-Rad), based on the Bradford dye binding procedure, and albumin was used as a standard. Each condition was performed in triplicate and expressed as OD 405 nm/µg protein.

### Phosphate measurement

Extra- and intracellular phosphate concentration was determined by Phosphate Colorimetric Assay Kit (BioVision). Extracellular phosphate was measured from the culture medium. Cells were treated with 0.1 M NaOH and 0.1% SDS to measure intracellular phosphate. Quantification of phosphate was measured with an OD at 650 nm by spectrophotometry. The results were normalized to the total protein concentration, which was measured in the cultures using a protein assay reagent (Bio-Rad), based on the Bradford dye binding procedure, and albumin was used as a standard. Each condition was performed in triplicate and expressed as nmol phosphate/µg protein.

### Cell proliferation assay

Cell proliferation of HAoSMCs with overexpression of sclerostin and mock was analyzed with 3-(4,5-Dimethylthiazol-2-yl)-2,5-diphenyltetrazolium bromide (MTT) assay. Cells were seeded in a 96-well plate (250 cells/100µL per well) in calcified condition for 10 days in standard condition and performed cell proliferation assay every 2 days. After the different times have elapsed, 10 µL of MTT (5 mg/mL) was added and the plate was incubated for 6 h at standard condition. Subsequently, 100 µL of lysis buffer (20% SDS in 50% formamide, pH 4.7) was added, and the plate was kept under standard condition overnight. Cell proliferation was measured with an OD at 570 nm by spectrophotometry. Four replicates per condition were carried out and were corrected by cell free media.

### Cell apoptosis assay

The percentages of apoptotic HAoSMCs with overexpression of sclerostin and mock in calcified condition were analyzed using a FITC Annexin V Apoptosis Detection Kit (BD Biosciences). Cells were washed with PBS twice and 10^5^ cells/100µL were incubated with Annexin V-FITC and propidium iodide at room temperature and in darkness for 15 min per condition. The samples were analyzed using a BD FACSAria IIIu flow cytometer (Becton Dickinson, BD Biosciences). The percentage of apoptosis was calculated by considering the sum of percentages of apoptotic cells (Annexin-FITC+/PI−) and late apoptotic cells (Annexin-FITC+/PI+). Each condition was performed in duplicate.

### Statistical analysis

Analyses were performed using SPSS version 28.0 software (SPSS, Inc., Chicago, IL) and GraphPad Prism v7.03 (GraphPad Software). Data were expressed as means ± standard deviation (SD) for variables normally distributed and as median with the interquartile range (IQR) for variables not normally distributed. The data for categorical variables were presented as percentages. A Kolmogorov-Smirnov test was used to test the normality of distribution of the continuous variables. The mean values between groups were compared using the unpaired Student´s t-test for continuous and normally distributed variables. The Mann-Whitney U test was used to compare variables not normally distributed. When the comparison between groups required an adjustment by covariates, a univariate analysis of covariance (ANCOVA) was performed. The χ^2^ test was used to compare categorical variables between groups. Associations between continuous variables were described by Spearman’s correlation coefficients. A multiple lineal regression model was performed to determine the variables independently associated with sclerostin (dependent variable), including the quantitative and qualitative variables linked in the bivariate analysis, and other variables biologically associated to sclerostin as independent variables. Data were expressed as B; 95% confidence interval (CI) (lower limit/upper limit). To identify sclerostin as an independent predictor of CVD, a multiple logistic regression model was performed, including prevalent CVD as a dependent variable. Statistical significance was set at *p* < 0.05 (two tailed) and *p* < 0.10 for multiple linear and logistic regression analysis. The independent variables included in the model were the established cardiovascular risk in addition to sclerostin levels. The usefulness of serum sclerostin as an estimator of CVD risk was assessed using a receiver operating characteristic curve (ROC). The area under the curve (AUC) indicates the probability to predict an event. AUC values greater than 0.75 indicate a good predictive performance.

Immunohistochemistry was performed on vascular tissue to assess the expression of sclerostin. The mean number of sclerostin-positive cells, along with the standard error of the mean (SEM), was determined for the different groups as well as for the intima-media and adventitia layers. This analysis allowed us to evaluate potential variations in sclerostin expression. To compare the differences in sclerostin-positive cells between the two vascular tissue layers, Student´s t-test were employed, providing a statistical assessment of the significance of the observed variations.

Furthermore, in the in vitro analysis of HAoSMCs, data were presented as means ± SD. To determine differences between the groups, the unpaired Student´s t-test was used for mean value comparisons. This statistical approach enabled us to evaluate the significance of observed variations in the experimental data between different groups of HAoSMCs.

## Results

### Characteristics of the study population

Table 2 summarizes the baseline characteristics of the entire population consisting of healthy subjects and T2D patients. Both groups were comparable in age and sex. As expected, patients with T2D showed a significantly worse metabolic profile in terms of BMI, waist circumference, FPG, HbA1c, and lipid profile. In addition, T2D group showed a significant increase in serum sclerostin level compared to control group (*p* = 0.003).

**Table 2:** Comparison of baseline characteristics between the control and T2D groups

Baseline Characteristics	Control	T2D	*p*
Men/women (n)	61/53	78/61	0.989
Age (years)	65 ± 9	65 ± 8	0.267
Body weight (kg)	74.9 ± 14.6	86.2 ± 14.4	< 0.001*
Height (cm)	163 ± 0.1	164 ± 0.09	0.298
BMI (kg/m^2^)	28 ± 4.8	31.8 ± 4.6	< 0.001*
Waist circumference (cm)	97 ± 10.7	105.9 ± 10.8	< 0.001*
FPG (mg/dL)	91 (84–99)	143 (107–173)	< 0.001*
HbA1c (%)	5.6 (5.4–5.8)	7.6 (6.9–8.6)	< 0.001*
TG (mg/dL)	103 (78–144)	139 (99–197)	< 0.001*
HDL-c (mg/dL)	54 ± 12	46 ± 11	< 0.001*
LDL-c (mg/dL)	116 ± 32	93 ± 40	< 0.001*
eGFR (mL/min/1.73 m^2^)	86.3 (73.5–94)	87 (71.1–97.5)	0.568
Calcium (mg/dL)	9.7 (9.5–10.1)	9.7 (9.5–9.9)	0.553
Phosphorous (mg/dL)	3.2 (2.9–3.5)	3.3 (2.9–3.6)	0.169
25(OH)D (ng/mL)	25.6 ± 8.5	20.7 ± 8.5	< 0.001*
P1NP (ng/mL)	43.8 ± 21	37.2 ± 15	0.002*
ALP (µg/L)	12.6 (10.3–16.9)	16.6 (13.3–21.4)	< 0.001*
iPTH (pg/mL)	52 (40.1–69.3)	46.8 (31.25-61)	0.007*
OC (ng/mL)	21.7 ± 17.4	10.8 ± 5.6	< 0.001*
Periostin (pmol/L)	1208.8 (972–1452)	1147 (936–1546)	0.975
Sclerostin (pmol/L)	32.6 (24.82–43.14)	39.02 (28.20-49.47)	0.003*

T2D, type 2 diabetes; BMI, body mass index; FPG, fasting plasma glucose; HbA1c, glycated haemoglobin; TG, triglycerides; HDL-c, high-density lipoprotein cholesterol; LDL-c, low density lipoprotein cholesterol; eGFR, estimated glomerular filtration rate; 25(OH)D, 25-hydroxyvitamin D; P1NP, procollagen type 1 N-terminal propeptide; ALP, alkaline phosphatase; iPTH, intact parathormone; OC, osteocalcin. Data for continuous and normally distributed variables are presented as mean ± standard deviation. Data for continuous variables not normally distributed, are presented as median followed by interquartile range in brackets. Data for categorical variables are presented as percentages. Student´s t-test and Mann-Whitney U test were used for comparisons of continuous and normally or not normally distributed variables, respectively, between groups. χ^2^ test was used for comparison of categorical variables between groups. * = *p* < 0.05 between groups

Furthermore, clinical, anthropometric, biochemical and bone parameters of the T2D participants according to the absence or presence of CVD are summarized in Table 3. In terms of clinical evaluation, notable variations were observed in CVD-defining factors, including hypertension, dyslipidaemia, and duration of diabetes. Additionally, significant differences were observed between the groups with respect to sex and age, indicating potential demographic influences on CVD development (Table 3). On the other hand, there were no significant differences between the groups in weight, height, BMI, or waist circumference (Table 3). Most biochemical parameters exhibited similar values between the two groups, except for HDL-c, LDL-c, eGFR, and calcium levels (Table 3). These variations suggest potential associations between these parameters and the presence of CVD in T2D patients. Furthermore, notable discrepancies were observed between the groups in terms of serum levels of proteins involved in bone metabolism, specifically periostin and sclerostin (Table 3). These findings indicate potential links between altered bone metabolism and the development of CVD in T2D patients.

**Table 3:** Intergroup comparison for T2D patients according to the presence of CVD.

	T2D without CVD	T2D with CVD	*p*
Men/women (n)	41/50	37/11	< 0.001*
Age (years)	65 ± 8	67 ± 7	0.040 *
**Clinical Evaluation**			
Body weight (kg)	86.51 ± 14.05	85.70 ± 15.18	0.377
Height (cm)	164 ± 0.09	166 ± 0.09	0.132
BMI (kg/m^2^)	32.14 ± 4.67	31.11 ± 4.49	0.107
Waist circumference (cm)	106.32 ± 11.08	105.03 ± 10.24	0.268
Diabetes duration (years)	13.43 ± 8.57	17.17 ± 9.99	0.011*
Systolic blood pressure (mmHg)	135.16 ± 18.14	134.79 ± 16.39	0.454
Diastolic blood pressure (mmHg)	81.25 ± 9.00	75.60 ± 12.04	0.003*
Hypertension (%)	80.2	95.8	0.013*
Dyslipidemia (%)	83.5	97.9	0.011*
Coronary heart disease (%)		56.3	< 0.001*
Cerebrovascular disease (%)		27.1	< 0.001*
Peripheral artery disease (%)		35.4	< 0.001*
Nephropathy (%)	13.2	25	0.080
Smoker or ex-smoker (%)	40	54.2	0.111
Alcohol consumption (%)	14.4	18.4	0.510
Sedentarism (%)	13	21.1	0.263
Fractures (%)	12.2	16.7	0.470
Osteopenia (%)	42.1	40.9	0.898
Osteoporosis (%)	10.5	6.8	0.498
**Current Medication Use**			
Insulin (%)	67	79.2	0.133
Oral antidiabetic drugs (%)	33	20.8	0.133
**Biochemical Measurements**			
FPG (mg/dL)	145.99 ± 48.68	152.88 ± 56.88	0.228
HbA1c (%)	7.79 ± 1.26	7.89 ± 1.60	0.340
TG (mg/dL)	139 (107–205)	142.5 (91.5–183)	0.232
HDL-c (mg/dL)	45 (39–52)	41 (35.50–48)	0.012*
LDL-c (mg/dL)	91 (67–126)	75 (51–103)	0.004*
eGFR (mL/min/1.73 m^2^)	89.70 (75.90–99.50)	80.90(62.30-93.35)	0.016*
Calcium (mg/dL)	9.8 (9.5–9.9)	9.6 (9.35–9.8)	0.010*
Phosphorous (mg/dL)	3.3 (3-3.7)	3.3 (2.9–3.6)	0.398
25(OH)D (ng/mL)	20.82 ± 8.22	20.55 ± 9.19	0.431
P1NP (ng/mL)	38.19 ± 15.57	35.15 ± 13.58	0.138
ALP (µg/L)	16.60 (13.50–22.50)	16.65 (12.10–19.30)	0.424
CTX (ng/mL)	1.69 (1.10–2.50)	1.35 (0.83–2.21)	0.218
iPTH (pg/mL)	45.95 (32.5–56.5)	47.95 (29.4–65.6)	0.970
OC (ng/mL)	11.24 ± 5.97	10.04 ± 4.72	0.255
Periostin (pmol/L)	1101.79 (853.41-1407.14)	1368.30 (1078.22-1734.54)	0.002*
Sclerostin (pmol/L)	36.64 (26.02–47.05)	45.99 (35.24–62.13)	< 0.001*

T2D, type 2 diabetes; CVD, cardiovascular disease; BMI, body mass index; FPG, fasting plasma glucose; HbA1c, glycated haemoglobin; TG, triglycerides; HDL-c, high-density lipoprotein cholesterol; LDL-c, low density lipoprotein cholesterol; eGFR, estimated glomerular filtration rate; 25(OH)D, 25-hydroxyvitamin D; P1NP, procollagen type 1 N-terminal propeptide; ALP, alkaline phosphatase; CTX, carboxy-terminal crosslinked telopeptide of type I collagen; iPTH, intact parathormone; OC, osteocalcin. Data for continuous and normally distributed variables are presented as mean ± standard deviation. Data for continuous variables not normally distributed, are presented as median followed by interquartile range in brackets. Data for categorical variables are presented as percentages. Student´s t-test and Mann-Whitney U test were used for comparisons of continuous and normally or not normally distributed variables, respectively, between groups. χ^2^ test was used for comparison of categorical variables between groups. * = *p* < 0.05 between groups

### Influence of diabetes status, sex and CVD on serum sclerostin levels 

Serum sclerostin levels were significantly higher in T2D patients (n = 139, 56.1% males) than in control subjects (n = 121, 56.2% males) (39.02 (28.20-49.47) pmol/L *vs.* 32.60 (24.82–43.14) pmol/L, *p* = 0.003). When T2D patients and control subjects were further divided according to sex, the significative differences in serum sclerotin levels remained for both males (T2D patients: 45.99 (32.05–55.36) pmol/L *vs*. controls subjects: 35.65 (27.62–47.35) pmol/L, *p =* 0.031), and females (T2D patients: 36.64 (26.88–43.02) pmol/L *vs.* controls subjects: 29.08 (21.51–34.82) pmol/L, *p* = 0.012). We found serum sclerostin levels significantly higher in males than females in the T2D group (45.99 (32.05–55.36) pmol/L *vs.* 36.64 (26.88–43.02) pmol/L, *p* = 0.002) and healthy controls (35.65 (27.62–47.35) pmol/L *vs.* 29.08 (21.51–34.82) pmol/L, *p* < 0.001).

**Fig. 1:**
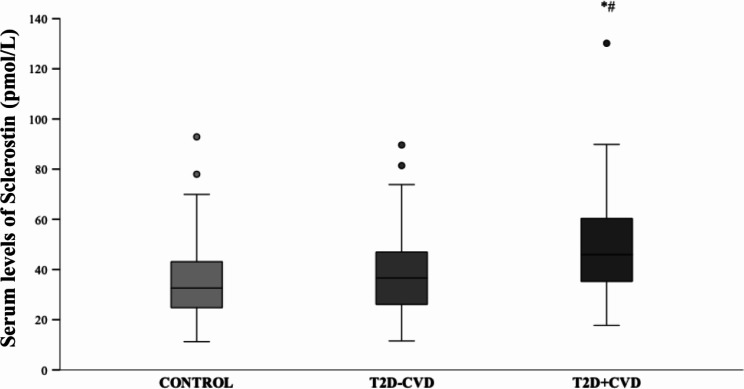
Box plot of serum sclerostin levels in controls (n = 121), T2D patients without CVD (n = 48), and T2D patients with CVD (n = 91). Box plot represents the minimum value, 25th percentile, median, 75th percentile, maximum value, and outliers for each group. The *p*-values between the different were performed by ANCOVA. * = *p* < 0.05 *vs. *Control; # = *p* < 0.05 *vs*. T2D without CVD. T2D, type 2 diabetes; CVD, cardiovascular disease.

The comparison of circulating sclerostin levels between healthy controls and T2D patients without CVD (n = 91, 45% males) and with CVD (n = 48, 77% males) revealed significant differences between groups (*p* < 0.001). The control group showed the lowest levels of circulating sclerostin levels , whereas T2D patients with CVD has the highest levels (*p* < 0.001). No significative differences were found between control group and T2D without CVD. After adjusting by age and sex, this trend in serum sclerostin levels remained unchanged among all groups (*p* < 0.001), except for control group *versus *T2D without CVD (*p =* 0.073) (Fig. 1). Specifically, our study revealed that T2D patients with peripheral arterial disease exhibited higher levels of sclerostin in serum compared to T2D patients without peripheral arterial disease.

### Determinants of serum sclerostin levels in the T2D patients

**Fig. 2:**
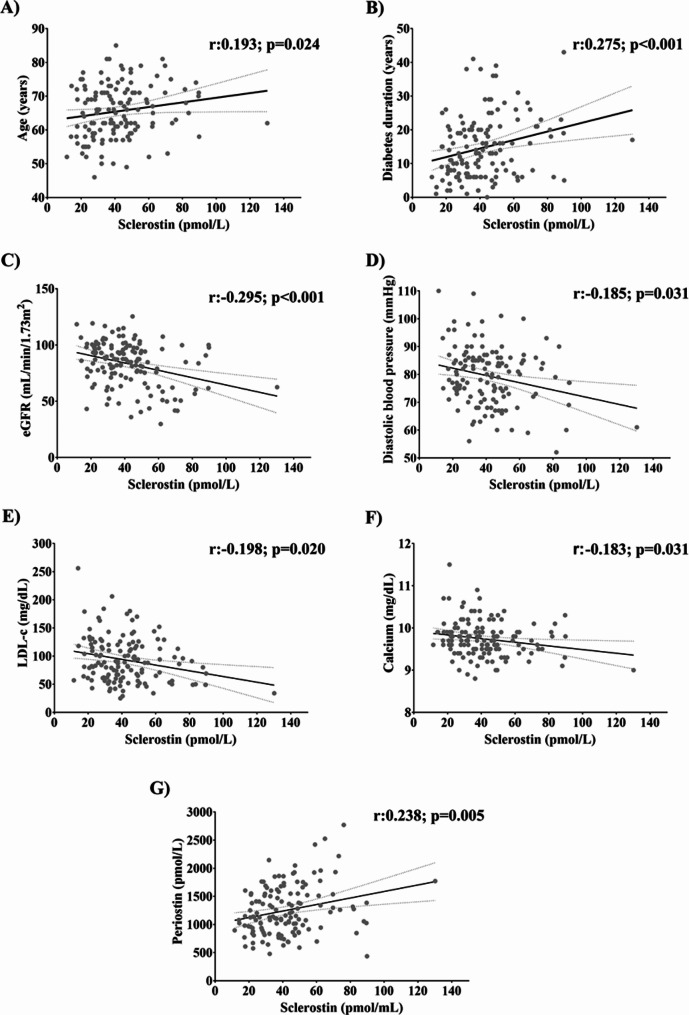
Scatter plots showing the correlation between sclerostin (pmol/L) and: **(A)** age (years), **(B)** diabetes duration (years), **(C)** eGFR (mL/min/1.73m^2^), **(D)** diastolic blood pressure (mmHg), **(E)** LDL-c (mg/dL), **(F)** calcium (mg/dL), and **(G)** periostin (pmol/L), in T2D patients (n = 139). The *p*-values between the different associations were performed by Spearman’s correlation coefficients (showing *p* < 0.05 in each scatter plot). eGFR, estimated glomerular filtration rate; LDL-c, low density lipoprotein cholesterol; T2D, type 2 diabetes.

We found a positive correlation between the circulating sclerostin level and CVD-defining factors such as age (r = 0.193; *p* = 0.024) and duration of diabetes (r = 0.275; *p* < 0.001). Whereas we found a negative correlation with eGFR (r=-0.295; *p* < 0.001), diastolic blood pressure (r=-0.185; *p* = 0.031), LDL-c (r=-0.198; *p* = 0.020), and calcium (r= -0.183; *p* = 0.031). In addition, we found a positive correlation between serum sclerostin levels and the bone marker periostin (r = 0.238; *p* = 0.005), in T2D patients (Fig. 2).

To investigate the factors influencing the level of sclerostin, a multiple linear regression analysis model was performed. The model included variables that were found to be associated with sclerostin based on prior bivariate analysis, including age, diabetes duration, eGFR, diastolic blood pressure, LDL-c, calcium, and periostin. Additionally, sex, current medication, and the presence of CVD were included as independent variables in the analysis. The results of this analysis will provide valuable insights into the multifactorial nature of sclerostin regulation and its associations with various clinical parameters in the context of our study population. The results showed that the variables independently associated with the serum sclerostin level were sex (B = 0.182; 95% CI [0.857/13.128]; *p* = 0.026), diabetes duration (B = 0.198; 95% CI [0.085/0.730]; *p* = 0.014), eGFR (B= -0.237; 95% CI [-0.388/-0.081]; *p* = 0.003), and presence of CVD (B = 0.176; 95% CI [0.483/13.689]; *p* = 0.036).

### Evaluating the sclerostin serum level as a potential indicator of CVD risk in T2D patients

Logistic regression modelling was performed to assess the variables related to CVD risk in T2D patients. The independent variables included in the model were age, sex, hypertension, dyslipidaemia, eGFR, sedentarism, tobacco use and years of diabetes duration, in addition to serum sclerostin level. We found that, in addition to sex (OR = 0.305; [0.120/0.771]; *p* = 0.012), hypertension (OR = 0.213; [0.043/1.069]; *p* = 0.060) and dyslipidaemia (OR = 0.142; [0.160/1.236]; *p* = 0.077), the serum sclerostin level was an independent estimator of CDV risk (OR = 1.026; [0.999/1.054]; *p* = 0.064) in T2D patients.

**Fig. 3:**
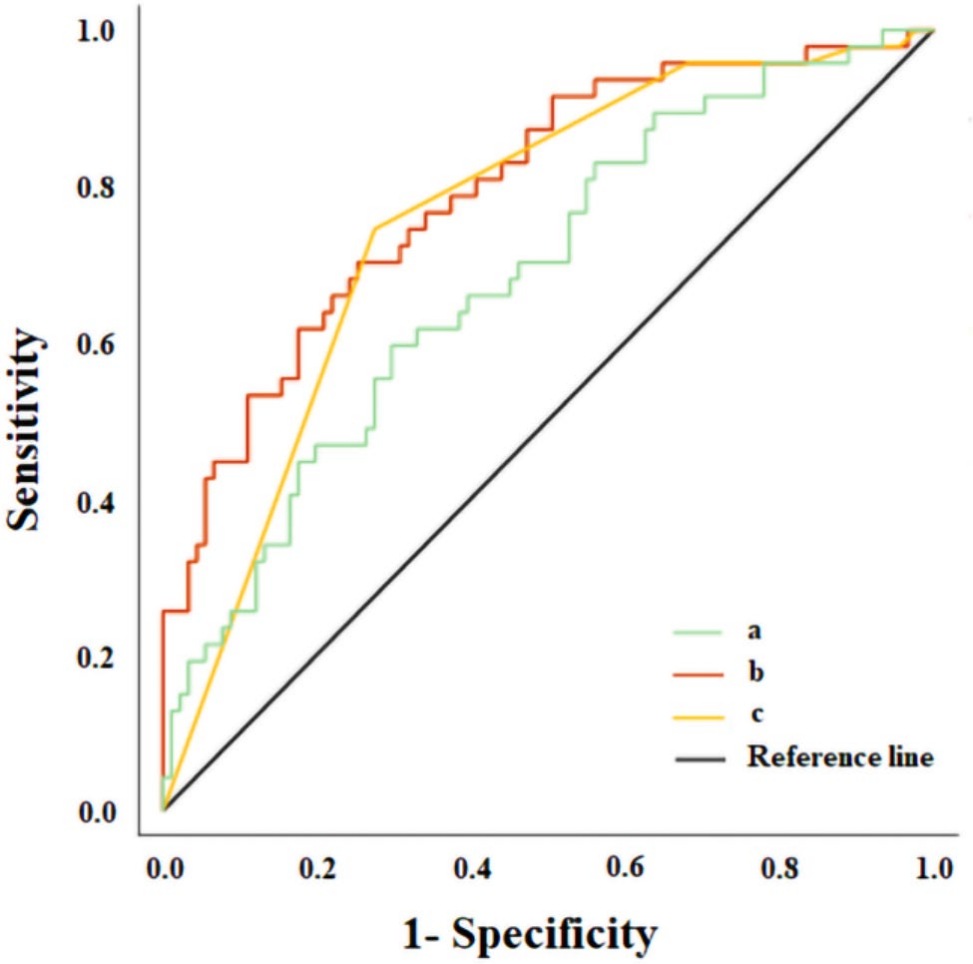
ROC curve for the usefulness of serum sclerostin level as an estimator of CVD in T2D patients (n = 139). **(a)** Serum sclerostin levels; AUC = 0.685; *p* = 0.003. **(b)** Age, sex, hypertension, dyslipidaemia, eGFR, sedentarism, tobacco use and years of diabetes duration and serum sclerostin level; AUC = 0.795; *p* < 0.001. **(c)** Age, sex, hypertension, dyslipidaemia, eGFR, sedentarism, tobacco use and years of diabetes duration; AUC = 0.757; *p* < 0.001. The independent variables included in the models were the established cardiovascular risk in addition to sclerostin levels. The usefulness of serum sclerostin as an estimator of CVD risk was assessed using a ROC curve. The AUC indicates the probability to predict an event and the values greater than 0.75 indicate a good predictive performance. ROC, receiver operating curve; eGFR, estimated glomerular filtration rate; T2D, type 2 diabetes; AUC, area under the curve.

To evaluate the predictive value of serum sclerostin level for CVD risk estimation, a ROC analysis was conducted. Two distinct models were assessed. The first model consisted of the main CVD risk factors, namely age, sex, hypertension, dyslipidaemia, eGFR, sedentarism, tobacco use, and years of diabetes duration. The second model included the same CVD risk factors along with serum sclerostin level as an additional variable. By comparing the performance of these two models, we aimed to determine the contribution of serum sclerostin level in improving the accuracy of CVD risk estimation. AUC of the model without sclerostin was 0.757; *p* < 0.001, whereas the AUC of the model including sclerostin was 0.795; *p* < 0.001 (Fig. 3).

### Sclerostin expression level in vascular tissue

**Fig. 4:**
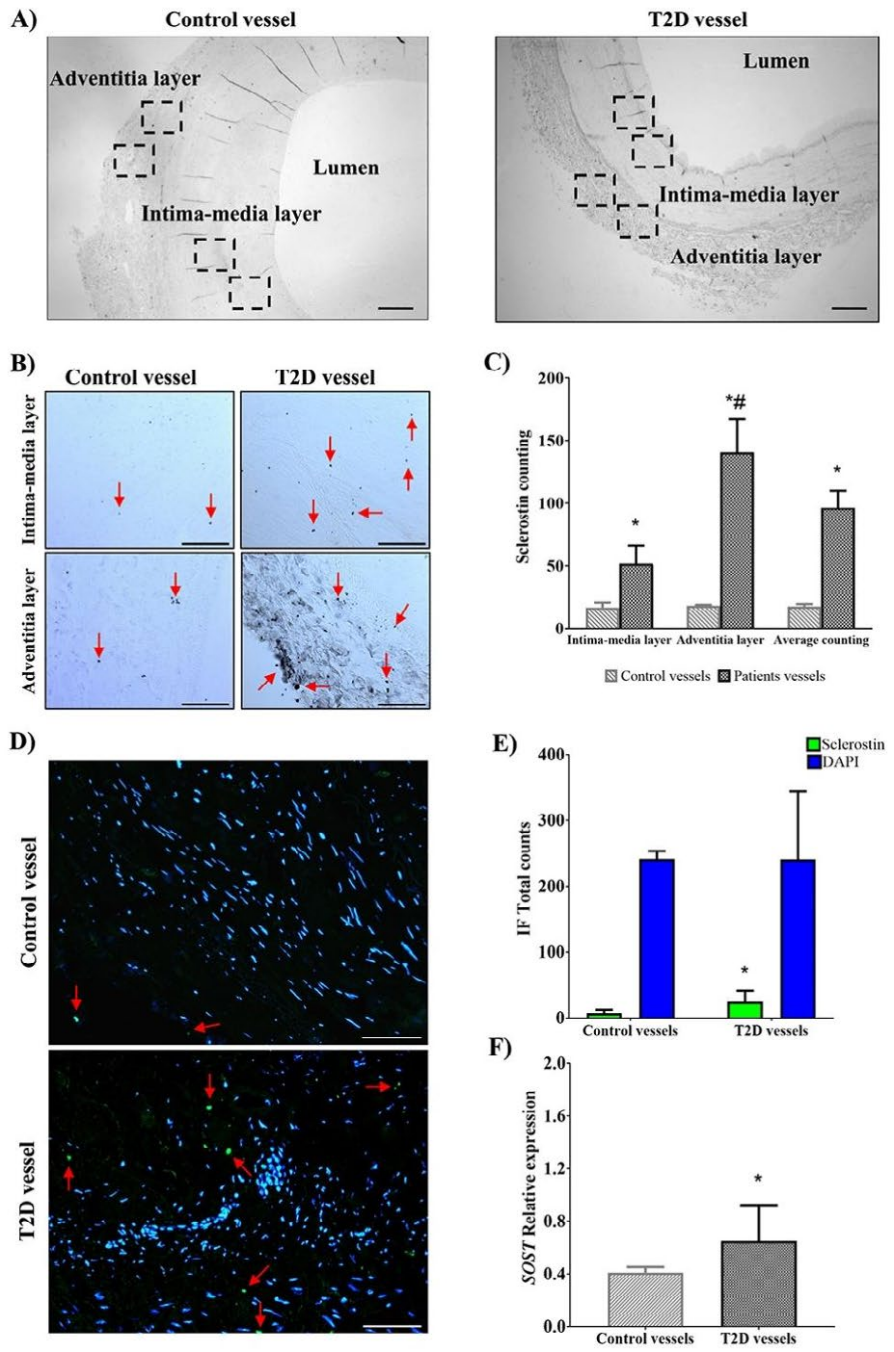
Sclerostin expression at vascular tissue. **(A)** Schematics of the immunohistochemistry microphotographs (2X) of the control subject vessel (left) and T2D patient vessel (right). All the microphotographs were captured following a medio-lateral axis in order to cover the two layers of the artery. The dashed boxes represent the schematic location of the microphotographs captured for the intima-media and the adventitia layer. (**B)** Representative microphotographs obtained at 20X magnification of the artery for the intima-media layer (top panel) and the adventitia layer of the artery (bottom panel) for T2D and control groups. Scalebars: 100µm. The red arrows show examples of sclerostin location. (**C)** Sclerostin count in calcified lower limb arteries of T2D patients (n = 7) and non-calcified arteries of control subjects (n = 3) by immunohistochemistry. Data are presented as the mean ± standard error of the mean of sclerostin labeled proteins in the intima-media and the adventitia layer of the artery and the total average for both groups. The *p*-values were determined by the unpaired Student´s t-test. * = *p* < 0.05 *vs.* control subjects’ vessels. # = *p* < 0.05 *vs.* intima-media layer of T2D patients’ vessels. **(D)** Representative microphotographs of immunofluorescence of sclerostin (green color), counterstained with DAPI (blue color) of control subject vessel (left) and T2D patient vessel (right). The red arrows show examples of sclerostin location. **(E)** Quantification of the immunofluorescence images of calcified lower limb arteries of T2D patients (n = 7) and non-calcified arteries of control subjects (n = 3). Data are presented as the mean ± standard error of the mean of sclerostin labeled proteins and DAPI in the total count of both groups. The *p*-values were determined by the unpaired Student´s t-test. * = *p* < 0.05 *vs*. control subjects’ vessels. **(F)** Sclerostin relative expression in both groups by RT-qPCR using the 2^−ΔΔCt^ method (fold-change). All data are presented as the mean ± standard error and Student´s t-test was used for the comparison. * = *p* < 0.05. T2D, type 2 diabetes; DAPI, 4’,6-diamidino-2-306 phenylindole; IF, immunofluorescence; *SOST*, sclerostin.

Immunohistochemistry and immunofluorescence were performed on calcified lower limb arteries of T2D patients (n = 7) and non-calcified arteries of healthy controls (n = 3). For immunohistochemistry, the analysis of the total average of sclerostin-positive cells revealed a significantly higher expression of sclerostin in T2D patients’ vessels compared to control subjects’ vessels (96.18 ± 13.61 *vs*. 17.25 ± 2.18, *p* = 0.003). When examining the location of sclerostin-positive cells, a significant increase was observed in both the intima-media layer (51.71 ± 14.19 *vs.* 16.50 ± 4.01, *p* = 0.025) and the adventitia layer (140.64 ± 26.59 *vs*. 18.00 ± 0.76, *p* = 0.010) in calcified vessels compared to healthy vessels. Furthermore, a higher expression of sclerostin was detected in the adventitia layer compared to the intima-media layer in calcified lower limb arteries of T2D patients (140.64 ± 26.59 vs. 51.71 ± 14.19, *p* = 0.006). However, no significant differences were observed between the layers in non-calcified vessels of healthy controls (18 ± 0.76 vs. 16.5 ± 4.01, *p* = 0.373) (Fig. 4C).

Immunofluorescence analysis revealed significantly increased sclerostin expression in T2D patients’ vessels compared to control subject’ vessels (24.4 ± 6.4 *vs.* 7.5 ± 2.8, *p* = 0.022) without significant differences in the total cell nuclei count between groups (*p* = 0.496) (Fig. 4E).

Additionally, the calcified lower limb arteries of T2D patients (n = 7) exhibited a significant upregulation of sclerostin mRNA compared to the non-calcified lower limb arteries of healthy controls (n = 3), with a 4.71-fold increase (*p* = 0.010) (Fig. 4F). These findings highlight the elevated expression of sclerostin in calcified lower limb arteries of T2D patients, both at the protein level and mRNA level, indicating its potential involvement in the pathogenesis of arterial calcification in T2D.

### Effect of sclerostin overexpression on mechanisms involved in calcification in HAoSMCs

To investigate the impact of sclerostin on HAoSMCs in a calcified medium, we employed a second-generation lentiviral packaging system to establish stable sclerostin overexpression in vitro. The effectiveness of sclerostin overexpression was confirmed through RT-qPCR. Remarkably, HAoSMCs transduced with *SOST* gene exhibited a significant 12,370-fold increase in sclerostin mRNA levels compared to the mock group, indicating successful and robust sclerostin overexpression (*p* < 0.001) under the same experimental conditions.

**Fig. 5:**
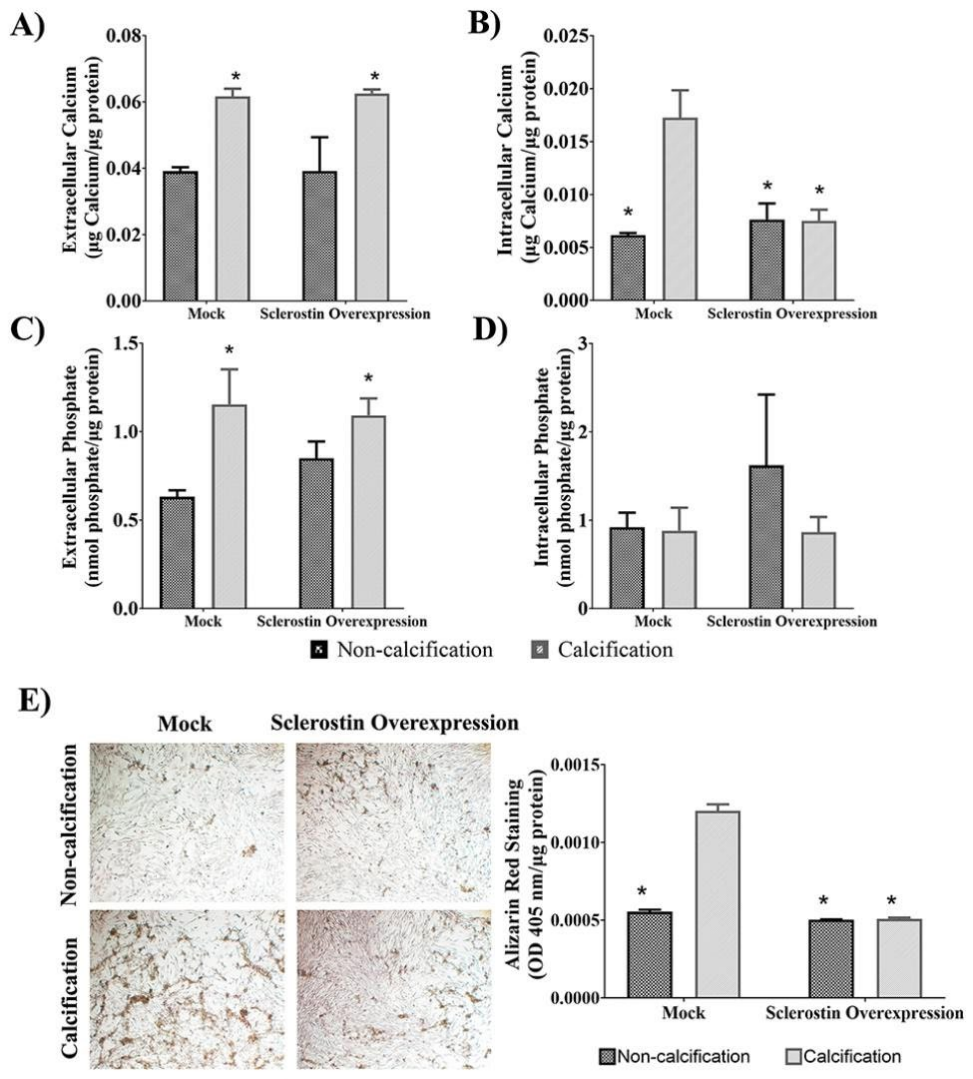
Calcium and phosphate determinations under different calcifying conditions in HAoSMCs. **(A) (B)** Extracellular and intracellular calcium concentrations (µg calcium/µg protein), **(C) (D)** extracellular and intracellular phosphate (nmol phosphate/µg protein). All determinations were measured in mock and HAoSMCs overexpressing sclerostin, under non-calcifying and calcifying conditions. **(E)** Representative microphotographs obtained at 5X magnification of Alizarin Red staining of mock and HAoSMCs overexpressing sclerostin, under both non-calcifying (top) and calcifying (bottom) conditions and normalized by total protein content. Scalebars: 200 μm. Calcium mineral depositions were analyzed in the different conditions and results were expressed as the OD 405 nm/µg protein; n = 3 biological replicates/group and n = 3 technical replicates/ biological replicates were performed. Data are represented as the mean ± standard deviation. The *p*-values between groups were determined by the unpaired Student´s t-test. In the figure A and C * = *p* < 0.05 *vs*. non-calcifying conditions. In figure B, * = *p* < 0.05 *vs*. calcified mock. HAoSMCs, Primary Human Aortic Smooth Muscle Cells; OD, optical density.

Extra- and intracellular calcium and phosphate concentration were measured in HAoSMCs with sclerostin overexpression and mock under calcification conditions. The normalized results showed that the extracellular calcium and phosphate concentrations in both mock and HAoSMCs with sclerostin overexpression under non-calcifying conditions is significantly lower than those of the cultures under calcified conditions (*p* < 0.05). The calcium and phosphate concentrations were similar in both groups ensuring proper calcification of the medium (Fig. 5AC). The results showed that HAoSMCs overexpressing sclerostin notably decrease the intracellular calcium concentration compared to mock under calcifying conditions (0.007±0.001 µg calcium/µg protein *vs. *0.017±0.003 µg calcium/µg protein; *p* < 0.001) revealing the calcification-inhibitory effect of sclerostin (Fig. 5B). However, no significant differences were observed in intracellular phosphate between mock and HAoSMCs with sclerostin overexpression under non-calcifying and calcifying conditions (*p* > 0.05) (Fig. 5D).

On the other hand, calcium mineral deposition was assessed by Alizarin Red staining followed the protocol of Alizarin Red S Staining Quantification Assay (ScienCell) and normalized by total protein content. The results showed decreased mineral deposits in HAoSMCs overexpressing sclerostin compared to mock under calcification conditions (0.0005±0.000008 OD 405 nm/µg protein *vs.* 0.0012±0.00004 OD 405 nm/µg protein; *p* < 0.001) (Fig. 5E), indicating the role of sclerostin in decreasing calcification.

**Fig. 6:**
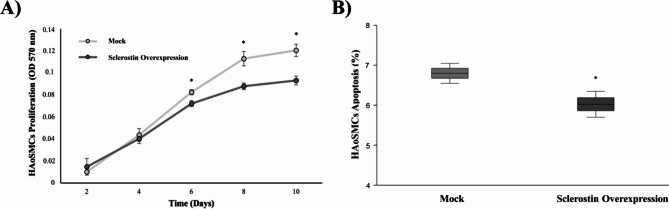
Effect of sclerostin overexpression on proliferation and apoptosis in HAoSMCs. **(A)** Effect of sclerostin overexpression on proliferation in HAoSMCs compared to mock (n = 4 biological replicates in each time/group). Each result was expressed as the OD at 570 nm. **(B)** Percentage of apoptosis in HAoSMCs with sclerostin overexpression compared to mock (n = 3 biological replicates/group and n = 2 technical replicates/ biological replicates). Data represent as the mean ± standard deviation of experiments performed. The *p*-values between groups were determined by the unpaired Student´s t-test. * = *p* < 0.05. HAoSMCs, Primary Human Aortic Smooth Muscle Cells; OD, optical density.

The impact of sclerostin overexpression on the proliferation and apoptosis of HAoSMCs was assessed. The MTT assay was used to assess the effect of sclerostin overexpression on HAoSMCs’ proliferation. The results demonstrated a significant decrease in the proliferation rate of HAoSMCs with sclerostin overexpression compared to the mock group. Specifically, at 6 days, there was a 12.5% reduction, at 8 days a 22.24% reduction, and at 10 days a 22.74% reduction (*p* < 0.001 for all conditions) (Fig. 6A). Furthermore, apoptosis-induced cell death was analyzed using annexin V and propidium iodide staining, followed by flow cytometry. Notably, HAoSMCs with sclerostin overexpression exhibited a significantly lower percentage of apoptosis compared to the mock group (6.02 ± 0.32% *vs*. 6.8 ± 0.25%, *p* = 0.015) (Fig. 6B).

**Fig. 7:**
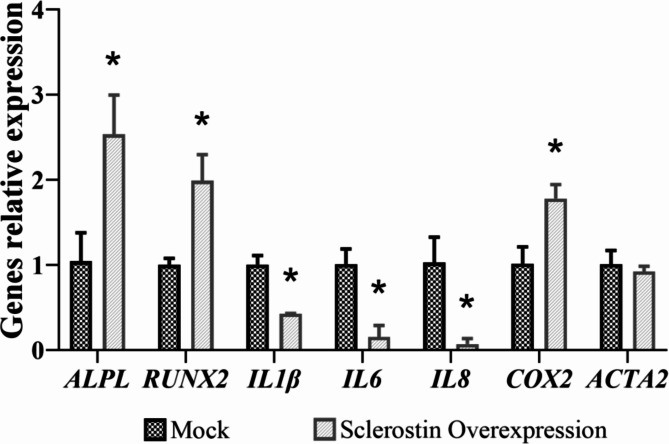
Relative expression of genes involved in bone metabolism, inflammation and contractility evaluated by qPCR in calcified HAoSMCs overexpressing sclerostin *vs.* calcified mock. Relative expression of each gene of interest was assessed using the 2^−ΔΔCt^ method. Data are represented as the mean ± standard deviation. The *p*-values between groups were determined by the unpaired Student´s t-test. * = *p* < 0.05. *ALPL*, alkaline phosphatase, biomineralization associated; *RUNX2*, runt-related transcription factor 2; *IL1β*, interleukin 1 beta; *IL6*, interleukin 6; *IL8*, interleukin 8; *COX2*, cyclooxygenase 2; *ACTA2*, actin aortic smooth muscle.

Additionally, the impact of sclerostin overexpression on the regulation of genes involved in bone metabolism, inflammation and contractility was evaluated by qPCR. The analysis revealed that HAoSMCs with sclerostin overexpression exhibited up-regulation of genes such as alkaline phosphatase, biomineralization associated *(ALPL)* encoding for phosphatase alkaline (ALP) (2.5-fold; *p* = 0.009), runt-related transcription factor 2 (*RUNX2*) (1.97-fold; *p* = 0.001) and cyclooxygenase 2 *(COX2*) (1.78-fold; *p* = 0.003), and down-regulation the genes such as interleukin 1 beta (*IL1β*) (0.43-fold; *p* = 0.005), interleukin 6 (*IL6*) (0.15-fold; *p* = 0.001) and interleukin 8 (*IL8*) (0.07-fold; *p* = 0.003). No significant differences were observed in the expression of actin aortic smooth muscle (*ACTA2*) encoding for α-smooth muscle actin (αSMA) (0.92-fold; *p* = 0.218) (Fig. 7). These findings provide valuable insights into the effects of sclerostin overexpression on HAoSMCs, indicating its role in inhibiting proliferation and promoting cell survival, as well as its potential influence on the regulation of genes associated with bone metabolism.

## Discussion

Our study examines for the first time whether sclerostin, a bone formation inhibitor protein, plays a detrimental or protective role in the development of atherosclerotic process in T2D population. Firstly, this study showed increased serum sclerostin levels in T2D patients with CVD compared to healthy controls (especially in males). Furthermore, higher serum sclerostin levels were independently associated with CVD in patients with T2D and significant correlations were found between serum sclerostin levels and cardiovascular risk factors such as age, diabetes duration, eGFR, LDL-c, calcium, diastolic blood pressure and periostin. Secondly, increased sclerostin expression was observed in calcified lower limb arteries of T2D patients compared to non-calcified vessels of control subjects. Thirdly, we found that sclerostin overexpression in VSMCs, in vitro, is involved in the decrease of calcium deposits in a calcified environment, as well as, in cell survival and in the regulation of the expression of different bone markers such as *ALPL* and *RUNX2* and inflammatory genes such as *IL1β*, *IL6* and *IL8*. Therefore, these findings suggest that the sclerostin increase may have a protective role on atherosclerosis development in T2D population.

The increased serum sclerostin levels observed in T2D patients with CVD are consistent with previous studies suggesting the potential role of sclerostin in vascular pathology. Some studies reported a positive correlation between serum sclerostin level and subclinical atherosclerosis [3], atherosclerotic lesions [14, 30] and cardiovascular mortality [17] in T2D population regardless of sex and age. Circulating sclerostin levels are generally higher among men, a trend that has been demonstrated in our study, both in T2D patients and control subjects. These results are supported by several studies in T2D patients [14, 15, 17] and healthy subjects [15, 31]. Mödder *et al.* reported than the larger skeletal size in men may explain the gender differences in circulating sclerostin production and release [31]. Regarding to age and in accordance with previously reported [31, 32], we found a positive association between serum sclerostin level and age that may be due to the skeletal remodeling or to imbalances in vascular remodeling associated with aging [14]. Furthermore, consistent with previous studies [3, 14], we found a significant positive correlation between serum sclerostin level and the duration of diabetes. The up-regulation of sclerostin in T2D patients could be produced by the hyperglycemia that has both a direct effect on bone cells and indirect effects through the formation of advanced glycation end-products affecting bone [15].

Based in previous studies, cardiovascular and renal alterations are closely related in T2D patients [33]. Several studies have reported higher serum sclerostin level in chronic kidney disease (CKD) patients with cardiovascular events [34], such as coronary [35] and aortic calcification [36] and the effect of sclerostin levels on CVD and all-cause cardiovascular mortality in patients with CKD [16, 37]. Accordingly, our results have shown a negative correlation between serum sclerostin level and eGFR observing more impaired renal function in patients with CVD and higher sclerostin levels. By contrast, some studies have described a positive association between higher levels of circulating sclerostin and better cardiovascular survival in dialysis patients [38, 39]. This discrepancy may be due to differences in patient populations with regard to age, comorbidities, duration of time on dialysis, and variability in the duration of the observational period.

Although the elevation of serum sclerostin level associated with cardiovascular alterations has been widely described, the function of this protein at the vascular level has not been studied in depth to date. Our biochemical results suggest a potential beneficial role of sclerostin on CVD in T2D patients due to its inverse association with some cardiovascular risk factors such as LDL-c, calcium, and diastolic blood pressure, which are considered the main factors contributing to susceptibility to atherosclerosis. It has been shown that sclerostin is stimulated in endothelial cells by pro-atherosclerotic factors including hyperglycemia, which increases the susceptibility of LDL-c to oxidation [33]. In T2D patients, oxidized LDL-c is efficiently recognized by scavenger receptors on macrophages that have accumulated within arterial walls, leading to lipid accumulation in arterial wall macrophages promotes atherogenesis and vascular stiffening [30]. This suggests that sclerostin could reduce lipid accumulation through LDL-c, thus decreasing vascular damage. Regarding calcium, our results suggest a protective role of sclerostin by inhibiting vascular calcification, since the activation of the canonical Wnt/b-catenin pathway releases calcium, activating the Wnt/Ca^2+^ route necessary for inflammatory process [18, 40]. These findings imply that elevated sclerostin levels in individuals T2D and CVD could contribute to the decrease in atherosclerotic plaque formation through the reduction of LDL-c and calcium levels. Consequently, this mechanism could potentially lead to a decline in blood pressure as we have observed. To our knowledge, this study is the first report revealing an association between serum sclerostin level with calcium and diastolic blood pressure in patients with T2D. However, future studies are required to corroborate these results. In addition, we found a significant positive correlation between serum sclerostin level and periostin, an extracellular matrix protein that is considered a biomarker for CVD [41] and it is involved in cardiac remodeling [42]. Some studies have shown an increase in serum periostin levels in diabetic vascular complications [42, 43]. The positive association observed between serum levels of periostin and sclerostin suggests that both proteins could play a protective role against the development of cardiovascular damage.   

Our results showed that the serum sclerostin level was an independent estimator of CVD risk in T2D patients. In this context, the literature has reported a positive association between circulating sclerostin levels and CVD [44], and indicates that sclerostin is a predictive marker of these pathologies [45]. Based on this, our ROC curve analysis reveals that the inclusion of serum sclerostin level , in addition to variables related to CVD development, improved the CVD risk prediction model.

Considering these finding and the fact that sclerostin acts as an inhibitor of bone formation, it is suggested that the increase in serum levels of this protein in patients who develop atherosclerotic processes could be a compensatory mechanism to block or attenuate the canonical Wnt/b-catenin pathway, with the aim of slowing vascular calcification. Although, studies have been carried out to elucidate the protective or pathological role of sclerostin at the vascular level in humans, all of them are merely observational. In this context, vascular effect of sclerostin has recently been of concern following the development of a new anti-osteoporotic treatment based on the monoclonal anti-sclerostin antibody, which simultaneously increases bone formation and, to a lesser extent, decreases bone resorption [18, 24]. This drug has shown a remarkable increase in bone formation and a reduction of fractures, although there are concerns regarding the degree of cardiovascular safety. A systematic review and meta-analysis report that treatment with anti-sclerostin antibody does not significantly increase the risk of composite cardiovascular outcomes [46]. However, significant cardiovascular adverse effects were reported during one study with anti-sclerostin antibody [23]. Moreover, the Active-Controlled Fracture Study in Postmenopausal Women With Osteoporosis at High Risk (ARCH) study, revealed an increased risk of serious adverse cardiovascular events in postmenopausal women during the first year treatment with anti-sclerostin antibody [25]. Currently, the existing data so far backs the notion of limiting the prescription guidelines outlined in the data sheet, which suggests that patients with a high risk of cardiovascular disease and stroke should not be eligible for treatment with anti-sclerostin antibody. Consistently, a study in animal models had described the potential protective role of sclerostin in vascular calcification. De Maré *et al.* showed evidence for a protective role of sclerostin during the development of vascular calcification by examining sclerostin expression in a mouse model of warfarin-induced vascular calcification. Serum levels and aortic expression of sclerostin were up-regulated in response to warfarin administration and increased vascular calcification was observed when warfarin was combined with anti-sclerostin antibody treatment [21].

Despite the clinical importance of sclerostin in vascular calcification, the precise biochemical processes that regulate this protein in this pathological process are not yet fully understood. Thus, it is necessary to develop experimental studies focused on discerning the potential protective role of sclerostin at vascular level. An essential step during the development of vascular calcification is the trans-differentiation of VSMCs to osteocyte-like cells capable of expressing typical osteocyte markers, including sclerostin [7]. The present study first established a cell model stably overexpressing sclerostin in HAoSMCs using a lentivirus system. This novel cell model offers a valuable tool for studying the role of sclerostin in VSMCs under calcification conditions.

Our study demonstrates that sclerostin overexpression leads to a reduction in intracellular calcium levels in HAoSMCs under a calcified environment. Intracellular calcium is required for the Wnt/Ca^2+^ pathway activated by Wnt5 and involved in endothelial inflammatory regulation [18, 40]. Notably, Wnt5 is expressed in human inflammatory diseases, including atherosclerotic plaques, and is expressed in VSMCs, supporting a pathophysiological role of Wnt5 in inflammatory regulation [40]. Moreover, in pulmonary arterial smooth muscle cells, an increase in the cytosolic calcium concentration is involved in physiological processes such as cell proliferation [47]. VSMCs switch from the contractile to the synthetic phenotype, facilitating proliferation as a physiological response induced by proinflammatory stimuli and oxidative stress for repair vascular damage in atherosclerosis [5, 6]. Increased calcium uptake has been shown to the phenotype switch to synthetic VSMCs and the development of vascular calcification [48]. Therefore, it is plausible to propose that the overexpression of sclerostin could play a role in decreasing the inflammatory response and inhibiting the proliferation of HAoSMCs, consequently leading to a reduction in atherosclerotic plaque formation. In this line, our results revealed a significant down-regulation in proinflammatory cytokines, such as *IL1β*, *IL6* and *IL8* in HAoSMCs overexpressing sclerostin compared to mock, suggesting a potential inhibitory role of sclerostin in inflammatory process in VSMCs. In addition, IL8 participates in the recruitment of neutrophils that adhere to and infiltrate the endothelial wall, favoring arterial stiffness [49]. Therefore, overexpression of sclerostin, in addition to reducing inflammation, would favor arterial elasticity via inhibition of these cytokines. In addition, quantitative alizarin red staining confirms that sclerostin overexpression reduces calcium deposition. We found less calcium deposition when sclerostin is overexpressed in HAoSMCs suggesting that this protein is acting as an inhibitor of calcification development. In this context, the scientific evidence has reported a role of COX2 in vascular calcification; however, there is controversy on its function with studies in opposite directions [50, 51]. The observed increased expression of *COX2* related to sclerostin overexpression showed in our results could have a protective role on vascular calcification supporting the beneficial function of sclerostin at vascular level. Agreeing, Cheng Gao *et al*., reported that COX2 may decrease the abnormal vascular calcification in humans [51]. These results support our hypothesis about the protective role of sclerostin in the atherosclerotic process.

Furthermore, our study reveals that sclerostin overexpression plays a role in regulating proliferation and apoptosis in HAoSMCs in calcified environment. The switch from contractile to synthetic phenotype promoting VMSCs proliferation has been identified as a crucial factor in the development of atherosclerotic plaque [52]. In response to vascular injury, VSMCs have been observed to significantly increase its rate of cell proliferation [5, 48], which implies an increase in collagen synthesis, further contributing to artery stiffening [30] and narrowing. While the proliferation rate of VSMCs may be elevated during the initial stages of lesion formation, it is not high in advanced mature lesion [48]. Specifically, we observed a decreased proliferation rate in HAoSMCs overexpressing sclerostin in a calcified medium. This reduction in proliferation rate could be attributed to the inhibitory effect of sclerostin on calcification, decreasing the intracellular calcium concentration, resulting in decreased proliferation, and consequently slowing the development of atherosclerotic plaque. Additionally, it is possible that the reduced proliferation in sclerostin overexpressing in HAoSMCs is influenced by the energy expenditure associated with the process of protein overexpression. On the other hand, there is extensive evidence that apoptosis of VSMCs can promote vascular calcification [53]. *In vitro*, apoptosis takes place before calcification occurs, and it is believed that apoptotic bodies contain elevated levels of calcium. These calcium-rich apoptotic bodies are subsequently deposited on the extracellular matrix, leading to the process of calcification. Furthermore, it has been shown that inhibition of apoptosis, for example by caspase inhibitors, significantly decrease both calcifying vesicle release and calcification [52]. Our results showed lower percentage of apoptosis in HAoSMCs with sclerostin overexpression. This finding suggests that the up-regulation of sclerostin could have a protective effect on HAoSMCs by reducing the formation of apoptotic calcium bodies, cell apoptosis and ultimately the calcification process. This observation aligns with the potential role of sclerostin in inhibiting calcification and promoting cell survival. These results provide evidence of the protective role of sclerostin in the development of CVD in the T2D population and confirm what was observed at both serum and tissue levels in these patients in our study and in previously described studies [3, 14].

This study has demonstrated the up-regulation of genes involved in bone formation, specifically *ALPL* and *RUNX2*, associated to sclerostin overexpression. ALP plays a crucial role in the production of inorganic phosphate, a significant molecule involved in calcification [6]. The induction of ALP in VSMCs implies an irreversible transformation towards calcified vascular cells [6]. As for *RUNX2*, a central transcriptional factor, is expressed by VSMCs to drive calcification [52]. It has been observed that cells expressing sclerostin also coexpress RUNX2 in calcified aortic valves [54]. Furthermore, it is noteworthy that the mineralization of VSMCs was associated by the up-regulation of key calcification genes, including *ALPL* and *RUNX2* [6]. Hence, we suggest that the up-regulation of *ALPL* and *RUNX2* could act as a compensatory mechanism in response to sclerostin overexpression that promotes inhibition of vascular calcification. Further research is necessary to elucidate the role of sclerostin in the up-regulation of genes involved in bone metabolism under calcified conditions.

At vascular level, our results revealed a significant increased expression of sclerostin in calcified artery of T2D patients both in the intima-media and adventitia layers compared to non-calcified vessels. *In vitro* results indicating that overexpression of sclerostin in calcified HAoSMCs leads to decreased intracellular calcium levels, calcium deposition, proliferation, and apoptosis, suggest that sclerostin plays a role in the intima-media layer of calcified artery of T2D patients reducing atherosclerotic plaque formation. Moreover, the increased sclerostin expression showed in the adventitia layer could be partly explained due to the expression of sclerostin in VSMCs from the intima-media layer could be transitioning to the adventitia layer to suppress the Wnt/b-catenin pathway limiting wall inflammation by decreasing inflammatory cytokines along with macrophage reduction [55] in the adventitia layer [56], thereby reducing arterial stiffness. However, future studies are required to elucidate the effect of increased sclerostin in the adventitial layer, as this is the first time that the increase of sclerostin has been described specifically in the adventitia layer of human arteries. These finding suggest that the elevation of serum sclerostin levels in T2D patients with CVD could be due to the increase of sclerostin at vascular level. According to our results sclerostin has been detected in the aorta of patients undergoing aortic valve replacement and is up-regulated in calcifying VSMCs and calcified valvular plaques compared non-calcified control valves [54]. Recently, sclerostin was identified in the media layer of VSMCs in plaques isolated from carotid arteries in subjects affected by severe vascular disease, irrespective of history of T2D [20].

These results at vascular levels, in conjunction with the outcomes derived from this research, indicate a potential protective function of sclerostin in the context of vascular calcification. However, it is important to note that further experimental studies are needed to validate this hypothesis in humans.

Our study presents some limitations. First, the cross-sectional design does not allow establishment of a cause-effect relationship. Moreover, our study population included only Caucasian individuals, from a specific area, and the use of common antihypertensive, antihyperlipidemic and antidiabetic drugs in patients may have influenced the results. Second, the number of vascular tissue samples from both controls and T2D patients with CVD is very small due to the difficulty of obtaining such samples, mainly for healthy controls, so these results should be interpreted with caution, therefore future investigations are necessary. However, our work has several strengths. Sclerostin has been evaluated both at the clinical level (serum and vascular tissue) and at the basic level (in vitro in VSMCs) in the same study establishing for the first time a cellular model that stably overexpressing sclerostin in HAoSMCs. Our cross-sectional study presents an exhaustive evaluation of clinical, anthropometric, and biochemical parameters, integrating all variables that could influence cardiovascular risk with experimental results. In addition, we performed rigorous statistical analyses, in order to obtain reliable results.

## Conclusions

We provide evidence supporting the protective role of sclerostin in the development of vascular calcification by reducing calcium deposition, decreasing proliferation and inflammation, and promoting cell survival associated with sclerostin overexpression. This suggest that sclerostin may mitigate the susceptibility to atherosclerosis by decreasing atherosclerotic plaque related to improved cardiovascular risk factors such as LDL-c, calcium, and diastolic blood pressure. These findings, both basic and clinical, contribute to the current understanding of the shared mechanisms between systemic bone and vascular physiology and pathology. Thus, our results emphasize the importance of considering the bone-vascular axis when designing therapeutic approaches for the treatment of impaired bone metabolism or vascular diseases.

## Data Availability

The datasets generated and/or analyzed during the current study are available from the corresponding author on reasonable request.

## Abbreviations

*ACTA2*: Actin, aortic smooth muscle
ALP: Alkaline phosphatase
*ALPL*: Alkaline phosphatase, biomineralization associated
BMI: Body mass index
CKD: Chronic kidney disease
*COX2*: Cyclooxygenase 2
CVD: Cardiovascular disease
eGFR: Estimated glomerular filtration rate
FPG: Fasting plasma glucose
HAoSMCs: Human primary aortic smooth muscle cells
HbA1c: Glycated haemoglobin
HDL-c: High-density lipoprotein cholesterol
HEK293T: Human embryonic kidney 293T cells
*IL1β*: Interleukin 1 beta
*IL6*: Interleukin 6
*IL8*: Interleukin 8
LDL-c: Lipoprotein cholesterol
*RPL13*: Ribosomal protein L13
*RUNX2*: Runt-related transcription factor 2
*SOST*: Sclerostin
T2D: Type 2 diabetes
TG: Triglycerides
VSMCs: Vascular smooth muscle cells
